# Debate: should we use variable adjusted life displays (VLAD) to identify variations in performance in general surgery?

**DOI:** 10.1186/s12893-015-0087-0

**Published:** 2015-08-28

**Authors:** Stephen O´Neill, Stephen J. Wigmore, Ewen M. Harrison

**Affiliations:** Department of Clinical Surgery, University of Edinburgh, Royal Infirmary of Edinburgh, Edinburgh, EH16 4SA UK

## Abstract

**Background:**

The recent push for the publication of individual surgeon outcomes underpins public interest in safer surgery. Conventional, retrospective assessment of surgical performance without continuous monitoring may lead to delays in identifying poor performance or recognition of practices that lead to be better than expected performance.

**Discussion:**

The variable life adjusted display (VLAD) is not new, yet is not widely utilised in General Surgery. Its construction is simple and if caveats are appreciated the interpretation is straightforward, allowing for continuous surveillance of surgical performance.

**Summary:**

While limitations in the detection of variations in performance are appreciated, the VLAD could represent a more useful tool for monitoring performance.

## Background

The recent push for the publication of individual surgeon outcomes underpins public interest in safer surgery. Conventional, retrospective assessment of surgical performance without continuous monitoring may lead to delays in identifying poor performance or recognition of practices that lead to be better than expected performance. The variable life adjusted display (VLAD) is not new, yet is not widely utilised in General Surgery. Its construction is simple and if caveats are appreciated the interpretation is straightforward, allowing for continuous surveillance of surgical performance. While limitations in the detection of variations in performance are appreciated, the VLAD could represent a more useful tool for monitoring performance.

## Discussion

### VLAD

The VLAD was established by Lovegrove et al. [1] to demonstrate the difference between observed and expected mortality over a specified period of time in Cardiac Surgery. The VLAD is sometimes called the expected-observed cumulative sum (CuSum) plot [2]. It is a graph that plots the cumulative difference in observed mortality from expected mortality on the y-axis against individual cases in the chronological order that they occur on the x-axis. Therefore a VLAD for a mortality rate that is equal to what is expected will end at zero, while a VLAD for a mortality rate above what is expected is seen as a falling line, and vice versa. This easily interpretable visual summary explains why the VLAD is popular amongst clinicians [3]. However, this apparent strength of the VLAD, can also be viewed as a weakness due to the strong temptation to view observed minus expected outcomes as ‘lives saved’ or ‘lives lost’, which is inappropriate.

### An example: expected mortality of 5 %

**Fig. 1:**
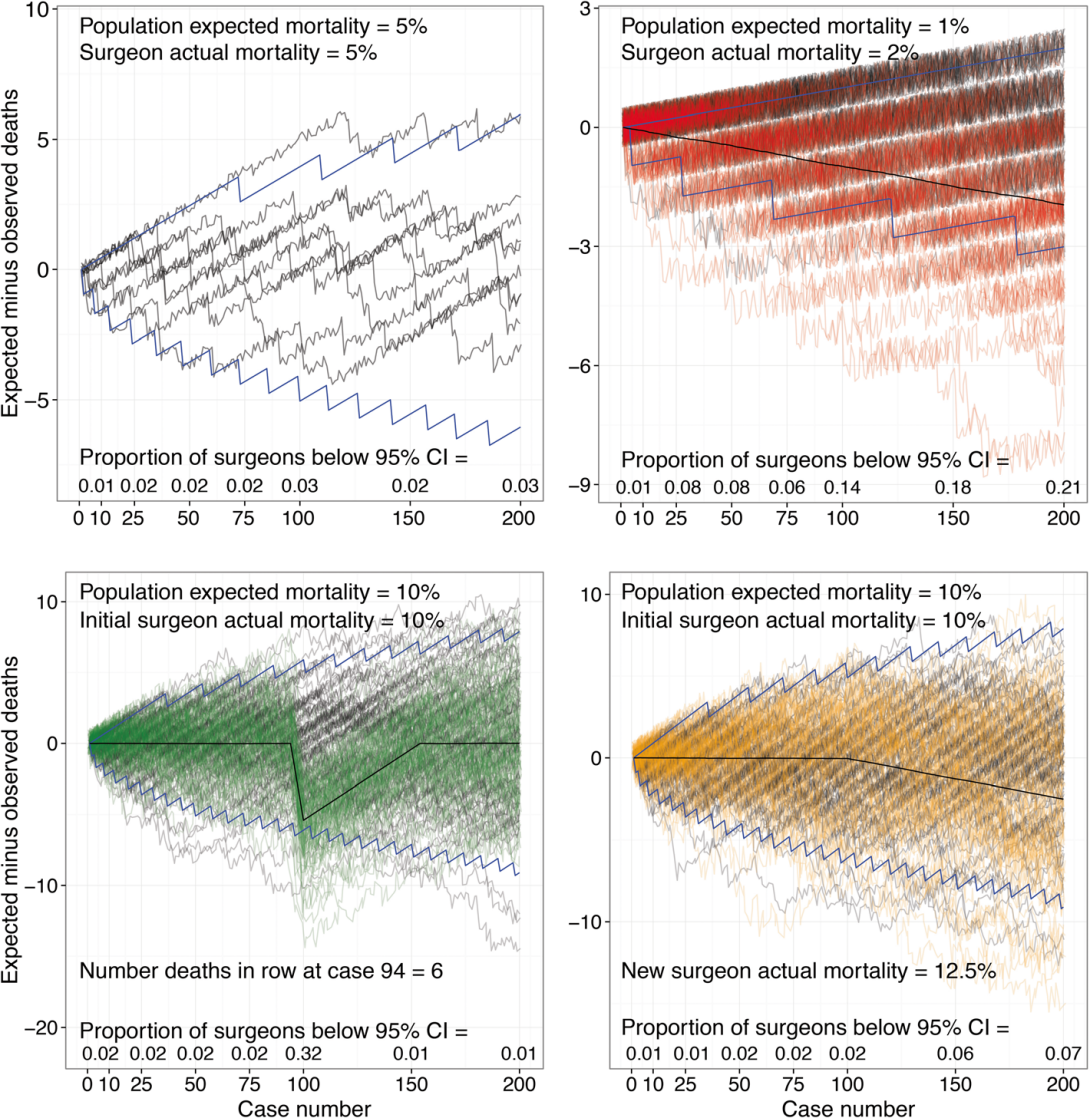
**a** VLAD for ten simulated surgeons (*black lines*) performing 200 cases with actual mortality equal to the population risk level of 5 %. The blue lines are 95 % control limits set for 10,000 similar plots. **b** 10,000 simulations of a VLAD for a surgeon with an actual mortality rate of 2 % (*red lines*, 200 shown) with a population risk level of 1 % (*black lines*, 200 shown). The mean is the thick black line and blue lines are 95 % control limits. **c** 10,000 simulations of a VLAD for a surgeon performing 200 cases with an actual mortality rate equal to the population risk level of 10 % for 94 cases but then having 6 deaths in a row before resuming their initial risk (*green lines*, 200 shown). The black lines (200 shown) are the population level risk of 10 %, the mean is the thick black line and blue lines are 95 % control limits. **d** 10,000 simulations of a VLAD for a surgeon performing 200 cases with an actual mortality rate equal to the population risk level of 10 % for 94 cases but then changing to an increased actual risk level of 12.5 % (*yellow lines*, 200 shown). The black lines (200 shown) are the population level risk of 10 %, the mean is the thick black line and blue lines are 95 % control limits. Plots available from: http://www.datasurg.net/vlad

Consider an example in a surgical context where the probability of death for a given procedure is 0.05 or 5 % (Fig. 1a).

Each consecutive procedure performed is assigned a binary value, which is 0 if there is no death and 1 if the patient died. A score is calculated from the predicted risk of death for that procedure, which in this example is 0.05. The VLAD score is calculated by subtracting the observed outcome (either 0 or 1) from the expected outcome (in this case 0.05). Therefore for a procedure resulting in a death the score would be 0.05 minus 1, which is equal to a downward increment of 0.95. While for a procedure that resulted in survival, the score would be 0.05 minus 0, which is equal to a positive increment of 0.05. If 20 cases were performed whereby the expected mortality and observed mortality was equal, then in the 19 cases where there was no death, the surgeon would have 19 upward increments of 0.05, which is equal to 0.95. This would be balanced by the one expected death that is observed, which would result in a downward increment of 0.95 and the line on the VLAD would return to zero. Therefore, in summary:

VLAD = Cumulative (Expected outcome - observed outcome)

Expected outcome is the probability of death e.g. 0.05

Observed outcome where survival = 0 and death = 1

### Advantages of VLAD

A VLAD is simple to construct and can be easily generated without any specialist statistical knowledge or software [4]. The VLAD facilitates targeted and continuous real time outcome surveillance. This allows the VLAD to include a surgeon’s entire caseload, which provides a better perspective of overall performance. Compared with the practice of retrospective assessment, this continuous surveillance mechanism offers the opportunity to identify and address the causes of unexpected results at an earlier stage. This may mitigate on-going poor performance or highlight better than expected performance [5]. Funnel plots are not designed for real time monitoring so the ability of the VLAD to be used as a continuous surveillance tool is a distinct advantage [3].

### Risk adjustments

When using the VLAD, an appropriate adjustment for operative risk is critical for ensuring accurate assessments. Defining a risk of death specific to each individual may be more robust than defining the same risk of death for all individuals undergoing one procedure. Outcomes are therefore adjusted for risk by different models that estimate the risk of death for each patient based on their individual characteristics and co-morbidities. However, caution must be observed in applying risk adjustments [3]. As surgical mortality rates decrease, risk scores need to be updated to represent the current standard of practice [3]. Tsang et al. [6] showed in paediatric cardiology how over a relatively short time period risk models could rapidly become out of date. No risk model is perfect and there may be inherent weaknesses in the method used to risk adjust. For example, the partial risk adjustment in surgery (PRAiS) model fails to adjust for certain co-morbid conditions and slightly underestimates risk for the highest risk patients. In a recent publication by Pagel et al. [7] this weakness in PRAiS led to a negative impression of performance in one UK centre that was involved in real time monitoring of risk-adjusted paediatric cardiac surgery outcomes using the VLAD.

### Control limits

The VLAD lacks control limits, which can make it difficult to assess the possible contribution of random variation to performance [8]. It also means that identifying the appropriate time to take action based on observed results is not quantitatively determined. This has led to criticism that the VLAD is limited in its ability to identify mortality rate changes with adequate speed [3]. However, since VLADs show the change in outcomes over time, one may not wish to wait to hit ‘significance’ before reflecting on an apparent trend. This approach could lead to the loss of lives that might have been saved, and irretrievable damage (maybe wrongly) to a surgeon’s career when some insight or retraining may have helped [9]. As such, the VLAD should not be considered a statistical evaluation [1].

Despite this, control limits, which are sometimes called rocket tails, can often applied to the VLAD to act as alert thresholds [8]. Walter A. Shewhart, the inventor of the industrial control chart technique, used three standard deviations control limits but in healthcare these control limits are often set at the 5 % level. Although this cut-off is arbitrary it can be considered as the point when the probability that differences between expected and observed outcomes are unlikely to be due to chance alone [8]. Nevertheless, as with any control limit, if control limits are applied to the VLAD, care needs to be taken, as apparent variation in performance may be highlighted when control limits are crossed simply as a result of random variation [8]. An often-cited analogy is the use of metal detectors to screen passengers at airports. In this situation the sensitivity of the detector can be varied. Low sensitivity runs the risk that a prohibited metal item such as a gun will pass undetected. High sensitivity reduces the risk of failing to detect a gun, but increases the number of passengers who are not carrying metal who will be pulled by chance out of line. Where the limits of detection should be set depend on the circumstances of the outcome, its seriousness and the need to detect outliers.

In Fig. 1a, typical VLADs were created by simulation using R for statistical programming (version version 3.1.1) for surgeons with an actual mortality rate exactly the same as that of the baseline risk across the entire population. Despite these surgeons working at the expected population mortality rate, there is apparent variation seen as a result of the process of random variation. It would therefore be expected in these VLADs that one surgeon in twenty may be above or below the 95 % control limit at any given time and therefore potentially subject to a review.

Using similar simulations, one may also consider the chance of a surgeon or unit with a mortality rate higher than expected being detected. This translates to the number of cases that require to be performed before the aberrant practice is identified. For example, with an expected mortality of 1 %, by 200 cases only 23 % of surgeons with an actual mortality rate of 2 % will have crossed a 95 % control limit (Fig. 1b). This focuses the mind as to what size of difference from normal practice should actually be considered different. We have created a web-application that can be used to explore these figures further (http://www.datasurg.net/vlad).

### Limitations of VLAD

Another criticism of the VLAD is that a good run of results may mask a subsequent poor run, which will mean that an excess of mortalities are needed to cross the control limit and trigger a review [5]. In Fig. 1c, surgeons are simulated with an actual mortality rate equivalent to that of the population mortality rate for 94 cases but then they have a poor run of 6 deaths in a row. Due to the previous good run, only 32 % of surgeons will cross the lower 95 % control interval control limit at this point.

There are also potential limitations with the VLAD for detecting more consistent changes in practice in an established system. This type of change may occur due to surgeon performance but could also potentially occur secondary to any significant change in the healthcare environment (e.g. critical care provision). In Fig. 1d, surgeons are simulated with an actual mortality rate equivalent to that of the population rate for 100 cases. At case 100, the actual mortality rate changes to a higher level, but this new “change point” is not detected given the wider control limits at this time. These figures can also be explored further using the aforementioned web-application (http://www.datasurg.net/vlad).

One method to prevent good runs masking subsequent poor performance is to prevent the VLAD from becoming positive so that only runs of worsening outcome are examined but this may lead to excess triggering and unneeded reviews of performance [5].

Alternative plots such as the risk-adjusted CuSum and risk-adjusted exponentially weighted moving average also overcome these limitations but may be more complex to construct. The risk-adjusted CuSum plot utilizes a sequential sampling technique to test the hypothesis that the risk of death is increased and doesn’t allow for accumulation of credit for good performance as the statistical test is bounded by the lower limit of zero. The risk-adjusted exponentially weighted moving average plot is a running estimate of the mean output of a process, where the most recent observations are given exponentially more weight than historically distant observations [10].

### Use of VLAD in General Surgery

Although it has taken time, examples of the use of VLADs in General Surgery are beginning to emerge. Collins et al. [3] retrospectively performed an analysis of the database of the Scottish Audit of Gastro-Oesophageal Cancer services using a VLAD. While Roberts et al. [5] recently published the first real-time, risk-adjusted VLAD of a single centre’s outcome after Ivor-Lewis oesophagectomy for oesophageal cancer. Guest et al. [4] applied the VLAD to single surgeon’s outcomes following oesophagogastric resections for cancer compared with those predicted by the Portsmouth predictor modification (P-POSSUM) score. Guest et al. [4] also went on to suggest that the VLAD was a potentially useful tool in the process of revalidation for surgeons. This could further extend the applicability of VLAD in the context in General Surgery, as could the use of the VLAD to monitor other performance outcomes such as post-operative complications. Even for the highest risk procedures in General Surgery (e.g. upper gastrointestinal cancer resection), the elective mortality rate is now on average <5 % [11]. Therefore other markers (e.g. failure to rescue, infection and anastomotic leak) could be particularly important in the General Surgical setting. However, before this can happen due consideration of data quality, definition of outcomes, case mix and institutional factors that affect outcome will be important.

## Summary

In efforts to improve patient safety the monitoring of surgical performance is becoming more widespread. As general surgery data will be increasingly placed in the public domain it is important that general surgeons take an active role in this process. Different methods of monitoring surgical performance need to be examined by the general surgical community and the use of VLADs could contribute significantly to identifying variations in performance.
